# Death of Judge Rowan

**Published:** 1843-08

**Authors:** 


### DEATH OF JUDGE ROWAN.

It is with no common degree of pain that we announce the demise of our venerable fellow-citizen, the Hon. John Rowan. He fell a victim to the prevailing epidemic, on the 13th inst., after an illness of two days. Judge Rowan was one of the founders of the Medical School of this city, and had presided over the institution from its origin.

This, of course, is not the place to speak of the virtues and the merits of the deceased; but we may be allowed to copy, from the proceedings of the Louisville Chancery Court, the following paragraph—part of a feeling tribute paid to his memory by those who had known him long and known him well.

"The deceased was born in Pennsylvania, and was brought to this country, with his father's family, when a small boy. He grew up with the State, and, at an early age, participated in her councils. He was a member of the Convention which formed the constitution of 1799; he was Secretary of State in 1804; he was elected to Congress from a district in which he did not reside in 1806; he was a member of the General Assembly many years; he was appointed Judge of the Court of Appeals in 1819; and, in 1824, he was elected to the Senate of the United States. The last public office he filled was that of commissioner for carrying out the late treaty with Mexico. He was a man of great literary acquirements—of full and varied information, and splendid eloquence. His genius was rich in original thought, beaming and striking, and, when called forth by a great subject, the boldness and sublimity of his conceptions were astonishing.

"His talents and learning made him the ornament of the bar; and he was, in all his intercourse, as full an exemplar of all that makes the bearing of a gentleman as any man who ever appeared in our courts."

